# Risk factors for persistent stress urinary incontinence after mid-urethral procedures

**Published:** 2008

**Authors:** Arun Chawla, Sreedhar Reddy, Joseph Thomas

**Affiliations:** Department of Urology, Kasturba Medical College, Manipal, India. E-mail: urologyarun@yahoo.com

## SUMMARY

This is a retrospective study of the postoperative outcome of using tension-free vaginal tape (TVT) and trans obturator tape (TOT) procedures in women with stress urinary incontinence (SUI). Analysis was done to identify factors that could predict persistence of incontinence after the two different procedures. There were 464 women in the age group of 25-80 years old who underwent TVT (252) or TOT (212) procedures. The post procedure follow-up ranged from six months to 52 months (mean 10.8). One hundred and fourteen patients in this study had preoperative urge urinary incontinence (UI) also which was not treated. Four hundred and eighteen patients had varying grades of cystocele which was not repaired during the sling procedures. The incidence of co-morbid conditions like diabetes, hypertension and bronchial asthma were comparable in both the groups. The urodynamic findings and mean degree of urethral hypermobility were similar in both the groups. The other baseline characteristics showed minor differences between the groups. The mean parity was higher in TVT, hysterectomy status was more in TOT and grade of cystocele was more in TVT. The severity of UI and amount of urinary leakage were slightly different in the two groups. Bladder was accidentally perforated in 12 patients (4.8%) in TVT. The rate of postoperative urinary retention in the TVT group was significantly higher than in the TOT group (15% *vs.* 6.6%). The overall cure rate of SUI was significantly higher in the TVT group than in the TOT group (92.1% *vs.* 84.9%). The authors concluded that the independent risk factors for the persistence of symptoms included the presence of co-morbid diseases (diabetes, hypertension, asthma), preoperative urge urinary incontinence, the higher grade of cystocele and the type of procedures (TVT or TOT).

## COMMENTS

There are only a few studies which try to identify factors associated with persistent UI after surgery. The cure rates in this study were significantly higher in the TVT group than in the TOT group. Latthe *et al.*, had concluded in their systematic meta-analysis that both techniques are not significantly different in their overall effectiveness.[1] The blind upward vaginal passage of suburethral tension-free vaginal tapes has been associated with a number of peri and postoperative complications including bowel, bladder, vascular and voiding dysfunction.[2] The complication of bladder perforations in this study was seen only in the TVT than TOT group. In comparison, the postoperative voiding dysfunction is much less in TOT than TVT as it takes the natural hammock shape. The reduced rate of voiding difficulty may appear to make TOT a preferred choice in patients with borderline flow rates.[3] In this series, patients with UI did not receive any medical management and none of the patients with cystocele underwent concomitant cystocele repair. This suggests that additional medical or surgical measures may contribute to the overall success of these procedures.

Independent risk factors for persistent leak after the two sling procedures were the presence of co-morbid diseases, preoperative urge urinary incontinence, associated severe grade cystocele and the type of corrective procedure carried out (TVT or TOT). This is a retrospective and non-randomized study, with the patient characteristics in both the groups quite dissimilar with a relatively short follow-up. High-quality randomized study with prospective design comparing the retropubic placement of mid-urethral sling with the two perineal approaches (TVT-O and TOT) will be more meaningful.

## References

[1] Latthe P, Foon R, Toozs-Hobson P (2007). Transobturator and retropubic tape procedures in stress urinary incontinence: A systematic review and meta analysis of effectiveness and complications. BIOG.

[2] Delorme E, Droupy S, de Tayrac R, Delmas V (2004). Transobturator tape: A new minimally-invasive procedure to treat female urinary incontinence. Eur Urol.

[3] de Leval J (2003). Novel surgical technique for the treatment of female stress urinary incontinence: transobturator vaginal tape inside -out. Eur Urol.

